# Sexual and reproductive health service utilization and associated factors among high school students in Ethiopia: systematic review and meta-analysis

**DOI:** 10.3389/frph.2024.1361662

**Published:** 2024-09-18

**Authors:** Amare Mebrat Delie, Ousman Adal, Abiyu Abadi Tareke, Eyob Ketema Bogale, Tadele Fentabel Anagaw, Misganaw Guadie Tiruneh, Eneyew Talie Fenta, Destaw Endeshaw

**Affiliations:** ^1^ Department of Public Health, College of Medicine and Health Sciences, Injibara University, Injibara, Ethiopia; ^2^ Department of Emergency, College of Medicine and Health Sciences, Bahir Dar University, Bahir Dar, Ethiopia; ^3^ SLL Project, COVID-19 Vaccine/EPI Technical Assistant at West Gondar Zonal Health Department, Amref Health Africa in Ethiopia, Gondar, Ethiopia; ^4^ Department of Health Promotion and Behavioral Science, School of Public Health, College of Medicine and Health Sciences, Bahir Dar University, Bahir Dar, Ethiopia; ^5^ Department of Health Systems and Policy, Institute of Public Health, College of Medicine and Health Sciences, University of Gondar, Gondar, Ethiopia; ^6^ Department of Adult Health Nursing, School of Health Sciences, College of Medicine and Health Sciences, Bahir Dar University, Bahir Dar, Ethiopia

**Keywords:** sexual and reproductive health, utilization, high school, students, Ethiopia, meta-analysis

## Abstract

**Introduction:**

Several studies have been done on the utilization of sexual and reproductive health services by high school students in Ethiopia, but they have yielded inconsistent results. This study aimed to evaluate the extent to which high school students in Ethiopia are using sexual and reproductive health services by conducting a systematic review and meta-analysis.

**Methods:**

Various electronic databases such as PubMed, Cochrane Library, AJOL, Google Scholar, and Grey Literature were used to search for relevant articles. Preferred Reporting Items for Systematic Reviews and Meta-Analysis Guidelines were followed for this review and meta-analysis. Heterogeneity was assessed using I^2^ and Cochrane Q statistical tests, and data analysis was done with STATA 17 software. Random effect meta-analyses were used to determine the overall utilization rate of sexual and reproductive health services.

**Result:**

This review included 20 studies with 12, 215 study participants. The pooled magnitude of sexual and reproductive health service utilization among high school students in Ethiopia was 29.79% (95% CI: 25.14, 34.43). Students with grades 11–12 (AOR = 2.33, 95% CI: 1.39, 3.90), aged between 20 and 24 years (AOR = 2.61; 95% CI: 1.79–3.81), having higher level of knowledge towards sexual and reproductive health issues (AOR = 3.10; 95% CI: 1.67–5.77), previous history of sexual intercourse (AOR = 4.18; 95% CI: 2.59–6.75), previous history of sexually transmitted infection (AOR = 3.74; 95% CI: 2.22–6.31), presence of a reproductive health service facility in the school (AOR = 2.55; 95% CI: 1.72–3.77), and ever-discussed reproductive health issues (AOR = 4.04; 95% CI: 1.62–10.03) were more likely to utilize sexual and reproductive health services.

**Conclusions:**

The overall utilization of sexual and reproductive services among high school students in Ethiopia was found to be low as compared to SDG 3.7. Older individuals with higher education levels and knowledge about sexual and reproductive health services, as well as those who have had previous sexual experiences or discussions about sexual health, are more likely to utilize reproductive health services. To increase utilization, the Ministry of Health and the Ministry of Education should prioritize these factors.

## Introduction

Sexual and reproductive health (SRH) refers to all conditions relating to the reproductive system's physical, mental, and social well-being and goes beyond simply being free from disease or infirmity (1). It includes the ability to be free from unwanted pregnancy, unsafe abortion, sexually transmitted infections, including HIV and AIDS, and all forms of sexual violence and coercion (1). SRH services include access to information and services in prevention, diagnosis, advice, treatment, and care, and ensure that all people can access services safely without having to travel long distances (2).

According to the World Health Organization, adolescents are considered to be between the ages of 10 and 19, while youth are between 15 and 24. The term “young people” encompasses individuals aged 10–24 (3). Approximately 1.2 billion of the world's total population are young people, and more than half of this population lives in developing countries (4). Sub-Saharan Africa (SSA) is a region where 23% of the total population (1.06 billion people) are adolescents (5). In Ethiopia, a SSA member state with a rapidly growing youth population, youth make up 33.8% of its estimated total population of 90 million (6). At this age, one of life's most rapid and complex stages occurs, characterized by significant physical, cognitive, behavioral, social, and psychological changes (7). Most people become sexually active during adolescence. Therefore, they will be exposed to a variety of SRH problems (1) due to poor decisions and actions (8). Young people are considered the country's greatest hope for the future, but their immaturity exposes them to certain risks, including unwanted pregnancies, sexually transmitted infections (STIs) such as HIV, and unsafe abortions (9). Based on a previous systematic review and meta-analysis conducted in Ethiopia revealed that the prevalence of risky sexual behavior among high school students was 28.13% (10).

According to the 2018 report of the Interagency Task Force on Reproductive Health, AIDS-related deaths among adolescents nearly tripled from 21,000 in 2000 to 60,000 in 2014, and AIDS-related deaths among women 1 in 4 gives birth before the age of 18, and 3.9 million people aged 15–19 undergo unsafe abortions each year (11). The 2016 Ethiopian Demographic and Health Survey (EDHS) National Report found that the adolescent birth rate was 80 per 1000 (6). According to the 2019 Performance Monitoring Action Survey, results from Ethiopia also revealed that the average age of the first sex is 16.4 years (12). Moreover, the 2019 Mini-EDHS found that only 36.4% of young women aged 15–19 years were using modern contraceptives (13). Despite the legal age of marriage in Ethiopia being 18, women typically marry at a much younger age than men. The median age for women to marry for the first time is 16.5 years, while for men it is 23.1 years. A large majority of women (58%) marry before they turn 18, compared to only 9% of men (14, 15). In this country, 13% of married teenagers between the ages of 15 and 19 have already started having children. Furthermore, the prevalence of HIV and AIDS among youth ages 15–24 is 0.34% in Harar (16).

A survey conducted at an Ethiopian higher education institution found that one-third of university students have had a previous history of sexual intercourse. Almost two-thirds of them were found to have already had a previous history of sexual intercourse before entering university, suggesting that SRH problems appear early and require intervention in early adolescence (17). SRH service utilization among high school students in Ethiopia varies widely across the country, from 18.4% (18) to 64.3% (19). Low utilization of sexual and reproductive health services affects adolescent health, impairs adolescent educational outcomes, increases dependency, and reduces a country's economic potential (20). Many social norms and practices that prevent sexually active youth from accessing contraceptives, maternity care, and other services based on age or gender pose challenges to effective service delivery (21, 22). The community was intolerant of premarital sex among adolescents and did not support the use of SRH or communication with unmarried adolescents (23). Both parents and teenagers view premarital sex as shameful and against their religious beliefs, particularly for girls as it could impact their future. Moreover, discussions about sexual health with unmarried teenagers are rare due to cultural taboos and fears of promoting sexual activity. The use of contraception is also frowned upon due to religious beliefs (23). Lack of sexual knowledge, lack of awareness of services, feelings of shyness and shame, fear of parents finding out about service use, and lack of confidentiality were associated factors for utilization of sexual and reproductive health services utilization (24).The National Adolescent and Youth Health Strategy (2021–2025) envisions achieving the following indicators by 2025: This includes reducing adolescent pregnancy rates from 12.5 to 7 and lowering the pregnancy-related mortality rate for those in the 15–19 age range from 0.39 to 0.29. It is also planned to increase the median age of first sex from 16.4 to 17 years, the median age for first marriages from 17.8 to 18 years, and the HIV prevalence among those aged 15–24 from 0.34% to 0.1% (16). Moreover, the World Health Organization's Sustainable Development Goal target 3.7 aimed to ensure that everyone has access to sexual and reproductive health-care services, including family planning, information, and education, and to incorporate reproductive health into national strategies and programs by 2030 (25). Despite initiatives, the prevalence of STIs, including HIV and AIDS (19.5%) is increasing, and abortion rates among students were 65 per 1,000 women, which is three times the national average for Ethiopia (26, 27). To facilitate the physiological, cognitive, emotional, and social transition of adolescents into adulthood, it is necessary to provide them with high-quality, reasonably priced SRH services. Particularly in underdeveloped nations, teenagers' SRH requirements are frequently neglected and do not currently receive enough attention (28). Improving the utilization of sexual and reproductive health services is the main strategy that lowers and prevents risks and issues related to adolescent reproductive health.

Although several studies were conducted on the magnitude and associated factors of sexual and reproductive health service utilization among high school students in Ethiopia, there was still no consistent evidence on the magnitude of sexual and reproductive health service utilization among high school students in Ethiopia. To make decisions in health programs, systematic review and meta-analysis studies are crucial with a high level of evidence. Therefore, this systematic review and meta-analysis were conducted to assess the magnitude and determinants of SRH service utilization among high school students in Ethiopia. This review will provide the first data regarding the pooled magnitude of SRH service among high school students in Ethiopia. The results of this review will close the evidence gap regarding the magnitude and contributing factors of SRH service among high school students in the nation. Thus, the review's findings will provide health policy planners and researchers with current data to aid in the development of suitable action plans aimed at enhancing the nation's sexual and reproductive health service utilization among high school students.

## Methods and materials

This review examines the literature on the extent and associated factors of sexual and reproductive health service utilization among secondary school students in Ethiopia. Therefore, the research questions for this review were: (i) “What is the overall utilization rate of sexual and reproductive health services among high school students in Ethiopia?” (ii) “What factors are associated with sexual and reproductive health services?”

### Information sources and search strategy

This systematic review and meta-analysis was conducted by the Prospective Reporting Items for Systematic Reviews and Meta-Analyses (PRISMA-2020) (29). PubMed, Cochrane Library, AJOL, Google Scholar, and Direct Google were used to search for articles. The search was conducted using search terms related to sexual and reproductive health services among high school students in Ethiopia. These were “secondary school”, “high school”, “preparatory school”, “grade 9–10”, “students”, “sexual and reproductive health service”, “youth-friendly”, “utilization”, “uptake”, “service”, “determinant”, “risk” and “associated factors” as well as “Ethiopia”. A comprehensive database search used the Boolean operators “OR”, “AND”, and MeSH terms.

### Eligibility

All original full-text English language research articles conducted in Ethiopia from December 2013 to December 2023 and published in peer-reviewed journals on sexual and reproductive health service utilization among Ethiopian high school students were included in this review. In addition, observational studies (cross-sectional, case-control, or cohort studies) were also included. In contrast, qualitative studies, surveys, editorials, reports, preprints, and studies that did not assess rates of SRH service utilization and associated factors were excluded from this study. Papers are screened for inclusion based on title, abstract, and other relevant information and then undergo a thorough evaluation before being included in the final review.

### Operational definitions

Sexual and reproductive health service use was defined as the use of any of the following SRH services: Sexual and reproductive health information, education and guidance, contraceptive services, pregnancy testing and care, voluntary counseling and testing (VCT), sexually transmitted infection (STI) screening, diagnostic and treatment services, and safe abortion care (30). Furthermore, from 9th grade to or his 12th grade, she or he is considered a high school student (31).

### Data extraction

After searching in relevant databases, the study was imported into Endnote version 20 and duplicates were removed. Then, three reviewers (AMD, MGT, ETF) downloaded the abstracts and screened them for eligibility. If reviewers disagreed about whether a search result was relevant to the study, it was included for retrieval. The relevance of the article was then assessed based on the article's title, topic, purpose, and methodology listed in the abstract. Abstracts were also assessed for compliance with the inclusion criteria. At this point, papers deemed irrelevant or outside the scope of the study were removed, the full texts of the remaining papers were downloaded for further analysis, and full-text reviews were excluded for reasons. Finally, after applying inclusion and exclusion criteria, eligible studies were exported to Microsoft Excel version 2019 using a standardized data extraction checklist. The following data were extracted: authors, year of publication, study design, study population, study setting, sample size, response rate, magnitude of SRHS utilization in percentage, and effect sizes of associated factors with the magnitude of SRHS utilization.

### Data quality assessment

The Joanna Briggs Institute (JBI) critical appraisal checklist was used to assess the quality of the studies. Using this tool as a protocol, reviewers (EKB, TFA, DE) assessed the quality of the original papers using a blind review approach. The average of ratings from three independent reviewers was used to decide whether an article should be included. Discrepancies in quality assessment results were resolved by another reviewer (OA), whenever appropriate. Those studies with scores of 5 or more in JBI criteria were considered to have good quality and were included in the review (32). Articles whose JBI criteria quality scores were less than 5; those studies that had methodological flaws, or incomplete reporting of results; or those for which full text was not available were excluded from the final analysis. Study researchers made two separate attempts to contact article authors whenever additional study information was needed (Table 1).

**Table 1:** Methodological quality assessment of included studies using the JBI critical appraisal checklist.

Study	Inclusion in the sample clearly defined	Study subjects and the setting described in detail	Exposure measured in a valid and reliable way	Objective, standard criteria for measurement of the condition?	Confounding factors identified	Strategies to deal with confounding factors stated	Outcomes measured in a valid and reliable way	Was appropriate statistical analysis used?	Total score
Binu et al.	No	Yes	Yes	Yes	Yes	Yes	Yes	Yes	7
Abate et al.	Yes	No	Yes	Yes	Yes	Yes	Yes	Yes	7
Teferi et al.	Yes	Yes	Yes	Yes	Yes	Yes	Yes	Yes	8
Aragie et al.	Yes	Yes	Yes	Yes	Yes	No	Yes	No	6
Abdurahman et al.	Yes	Yes	Yes	Yes	Yes	Yes	Not	Yes	7
Sertsu et al.	No	Yes	Yes	Yes	Yes	Yes	No	Yes	6
Tsegaw et al.	Yes	Yes	Yes	Yes	Yes	Yes	No	Yes	7
Yonas et al.	Yes	Yes	Yes	No	Yes	Yes	No	Yes	6
Demeke et al.	No	Yes	Yes	Yes	Yes	Yes	Yes	Yes	8
Simegn et al.	Yes	Yes	Yes	Yes	Yes	Yes	Yes	Yes	8
Gurara et al.	No	Yes	Yes	Yes	Yes	Yes	Yes	Yes	7
Fikadu et al.	Yes	Yes	Yes	No	Yes	Yes	No	Yes	6
Abebe et al.	Yes	Yes	No	No	Yes	Yes	No	Yes	5
Helamo et al.	Yes	Yes	No	No	Yes	Yes	No	Yes	5
Haile et al.	Yes	Yes	Yes	Yes	Yes	Yes	Yes	Yes	8
Bogale et al.	No	No	Yes	Yes	Yes	Yes	Yes	Yes	6
Wakjira et al.	Yes	No	Yes	Yes	Yes	Yes	Yes	Yes	8
Dina et al.	Yes	Yes	Yes	Yes	Yes	Yes	Yes	Yes	8
Bilal et al.	Yes	Yes	Yes	Yes	Yes	Yes	Yes	Yes	8
Geremew et al.	Yes	Yes	Yes	Yes	Yes	Yes	Yes	Yes	8

## Data analysis

Information on study characteristics from Microsoft Excel was exported to Stata software version 17 for further statistical analysis. Data were summarized by statistical tables, figures, and forest plots. A meta-analysis was conducted to determine the pooled extent of SRHS use and identify associated factors. The heterogeneity of study results was assessed using the I^2^ statistics (33) and Cochrane Q statistics (34). We used funnel plot asymmetry, Egger's, and Begg-Mazumdar Rank correlation tests to check for publication bias (35). In addition, we conducted subgroup analyses based on region and sample size. Sensitivity analyses were also conducted to detect the influence of each study on the overall pooled magnitude of SRHS utilization by excluding one study at a time.

## Results

**Figure 1:**
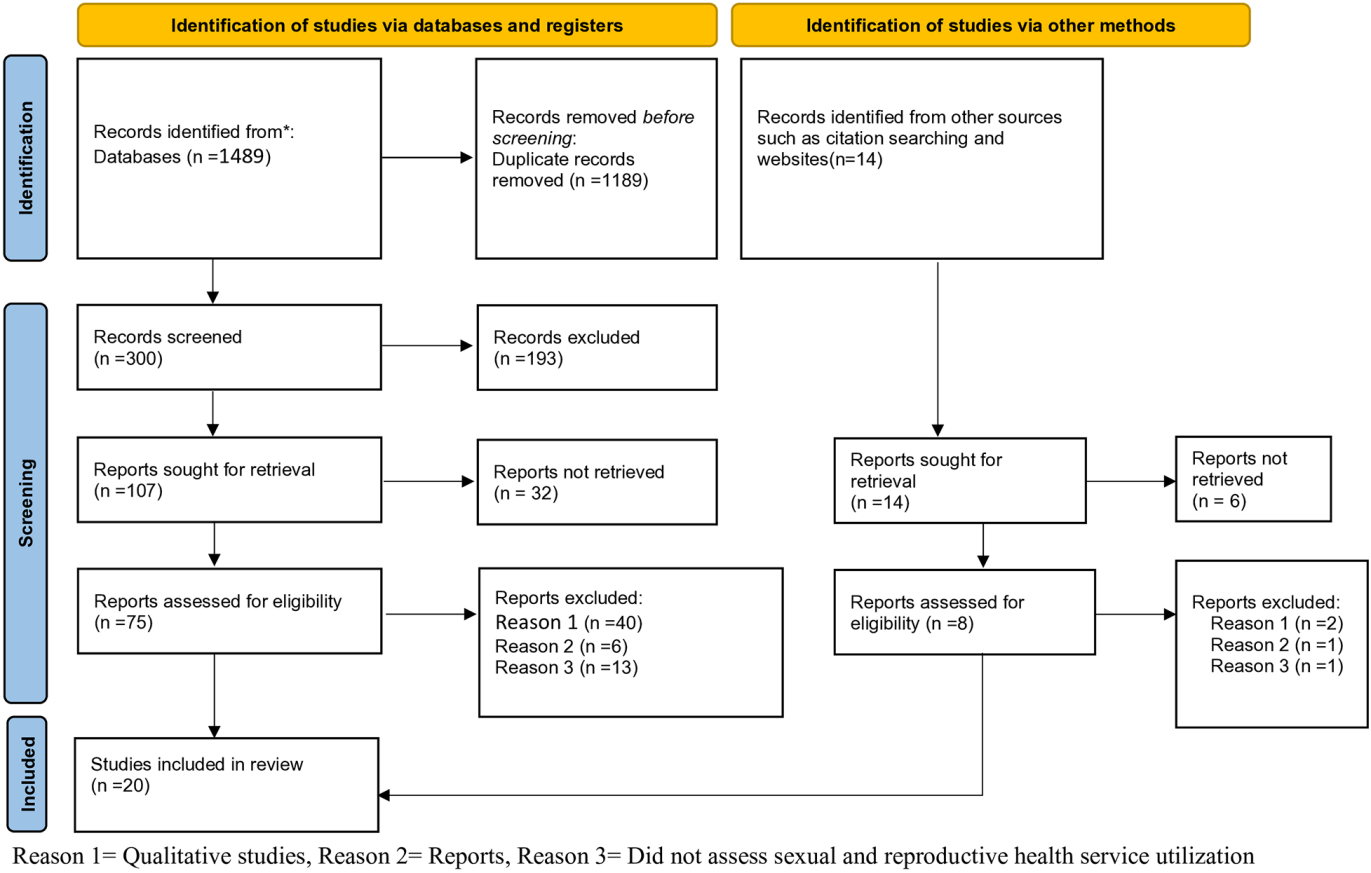
PRISM flow diagram of study selection of sexual and reproductive health service utilization among high school students in Ethiopia.

Through our initial database search, 1,503 records were located. 1,189 records were excluded due to duplicates, 231 articles out of 314 that were examined by title and abstract were removed since the study's area was outside of its acceptable limits and the people who participated in the study weren't secondary school students. For the full-text review, 83 articles were included. Finally, after applying inclusion and exclusion criteria, 20 studies were added to the final review (Figure 1).

### Study characteristics

In the current meta-analysis, 20 articles were included. A total of 12,215 high school students were involved to determine the pooled magnitude and associated factors of sexual and reproductive health utilization. Of 20 included studies; 18 used cross-sectional study design (18, 21, 36–51), 1 used cross-sectional study design complemented with qualitative inquiry (52) and the remaining 1 study used comparative cross-sectional study design (53). All of the included articles in this review were published between 2014 and 2023. The 20 studies were conducted in Tigray region (*n* = 1), Amhara region (*n* = 7), Oromia region (*n* = 5), Southern Nations, Nationalities, and Peoples' region (*n* = 4), Harari region (*n* = 1) and Benishangul Gumuz region (*n* = 1) of Ethiopia (Table 2).

**Table 2:** Summary of characteristics of included articles for systematic review for youth-friendly SRH service utilization among high school students in Ethiopia.

Serial number	Author with references	Publication year	Region	Study design	Study area	Study Population	Sample Size	Response Rate (%)	SRH service utilization (%)
1.	Binu et al. (30)	2018	Oromia	Cross-sectional	Nekemete town	Grade 9–12 students	768	96	21.2
2.	Abate et al. (48)	2019	Amhara	Cross-sectional	Woreta town	Grade 9–12 students	376	94	24.6
3.	Teferi et al. (38)	2022	SNNP	Cross-Sectional	Areka town	Grade 9–12 students	572	95.3	44.2
4.	Aragie et al. (46)	2021	Amhara	Cross-sectional	Woldia town	Grade 9–10	420	100	64.3
5.	Abdurahman et al. (47)	2022	Oromia	Cross-sectional	Haramia District	Grade 9–12 students	642	92.7	23.5
6.	Sertsu, et al. (40)	2023	Harari	Cross-sectional	Harari region	Grade 9–12 students	1275	97.6	25.3
7.	Tsegaw et al. (37)	2022	Amhara	Cross-sectional	East Belesa District	Grade 9–12 students	346	99.8	28.9
8.	Yonas et al. (36)	2022	SNNP	Cross-sectional	Dawro zone	Grade 9–12 students	835	98.8	26
9.	Demeke et al. (52)	2022	Amhara	Cross-sectional with qualitative inquiry	North Showa zone	Grade 11–12 students	596	98.5	32.7
10.	Simegn et al. (39)	2020	Amhara	Cross-sectional	Debretabor town	Grade 9–12 students	690	99.1%	28.8
11.	Gurara et al. (42)	2020	Oromia	Cross-sectional	Adama town	Grade 9–12 students	359	97.8	34
12.	Fikadu et al. (43)	2020	Oromia	Cross-sectional	Ambo town	Grade 9–12 students	376	100	20.7
13.	Abebe et al. (21)	2014	Amhara	Cross-sectional	Bahirdar city	Grade 9th and 11th students	818	100	32.2
14.	Helamo et al. (41)	2018	SNNP	Cross-sectional	Hadiya Zone	Grade 9–12 students	634	90.3	38.5
15.	Haile et al. (53)	2020	SNNP	Comparative cross-sectional	South Omo Zone	Grade 9–12 students	458	426	21.83
16.	Bogale et al. (44)	2020	Oromia	Cross-sectional	East Shewa zone	Grade 9–12 students	362	360	34.4
17.	Wakjira et al. (49)	2022	Oromia	Cross-sectional	Arsi Zone	Grade 9–12 students	800	96.74	26.1
18.	Dina et al. (50)	2021	Benishangul Gumuz	Cross-sectional	Assosa Zone	Grade 9–12 students	375	93.75	32.0
19.	Bilal et al. (51)	2014	Tigray	Cross-sectional	Mekelle- town	Grade 9–12 students	1,042	100	21.59
20.	Geremew et al. (18)	2018	Amhara	Cross-sectional	Mecha district	Grade 11–12 students	565	98.43	18.4

NB, note bene; SNNP, South Nations Nationalities Peoples of Ethiopia; SRH, sexual and reproductive health.

### The magnitude of sexual and reproductive health service utilization among high school students in Ethiopia

**Figure 2:**
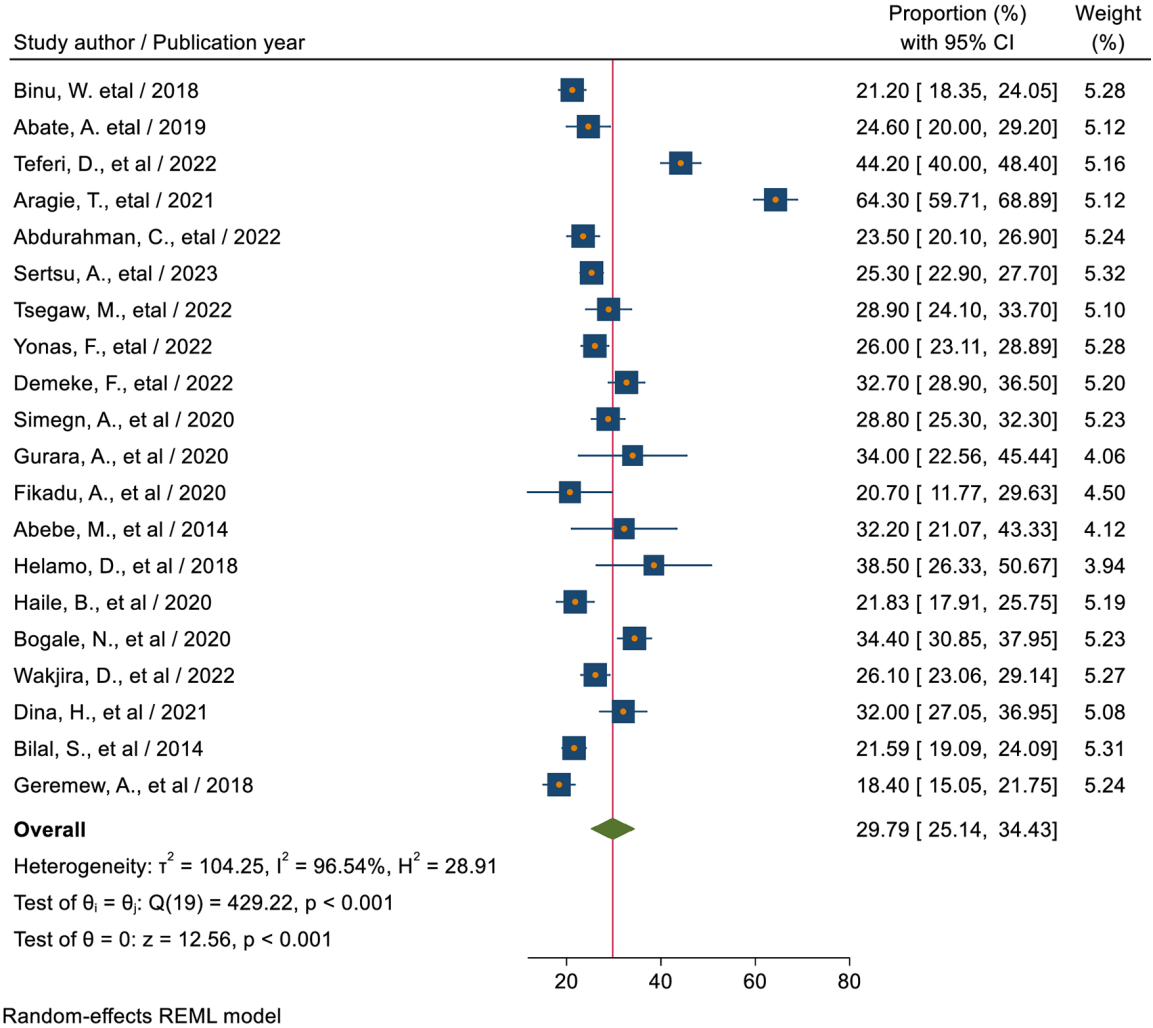
Forest plot for the pooled magnitude of sexual and reproductive health service utilization among high school students in Ethiopia.

The magnitude of sexual and reproductive health service utilization among high school students in Ethiopia, from included studies in this meta-analysis, ranged between 18.4% (18) to 64.3% (46). Based on the current meta-analysis, the pooled magnitude of SRHU among high school students in Ethiopia was 29.79% (95% CI: 25.14, 34.43). There was high and significant heterogeneity between studies (*I*^2^ = 96.54%; *P* < 0.01indicating great variability in the magnitude of SRHSU across studies; the random-effect model was used to estimate the pooled magnitude of SRHSU among high school students in Ethiopia (Figure 2).

#### Subgroup analysis based on region

**Figure 3:**
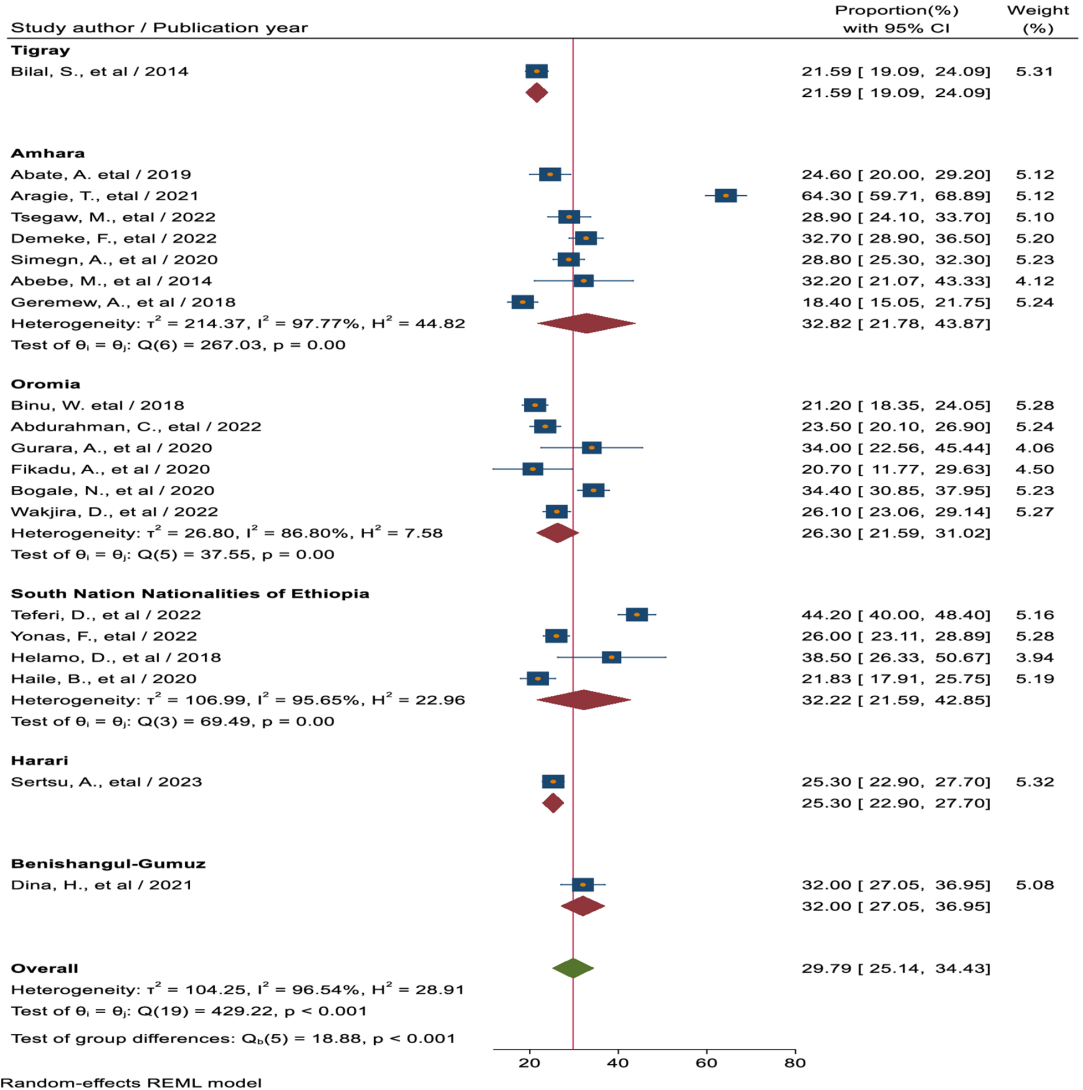
Forest plot for the pooled magnitude of sexual and reproductive health service utilization among high school students based on the region in Ethiopia.

Subgroup analysis was carried out in this meta-analysis according to the sample size and the country's study region. As a result, the Southern Nations, Nationalities, and Peoples' region had the largest magnitude of SRHSU (32.22%, 95% CI: 21.59, 42.85), followed by the Amhara region, reaching (32.82%, 95% CI: 21.78, 43.87), and the Oromia region (26.41%, 95% CI: 20.44, 32.38), which had the lowest magnitude (Figure 3).

#### Subgroup analysis based on sample size

**Figure 4:**
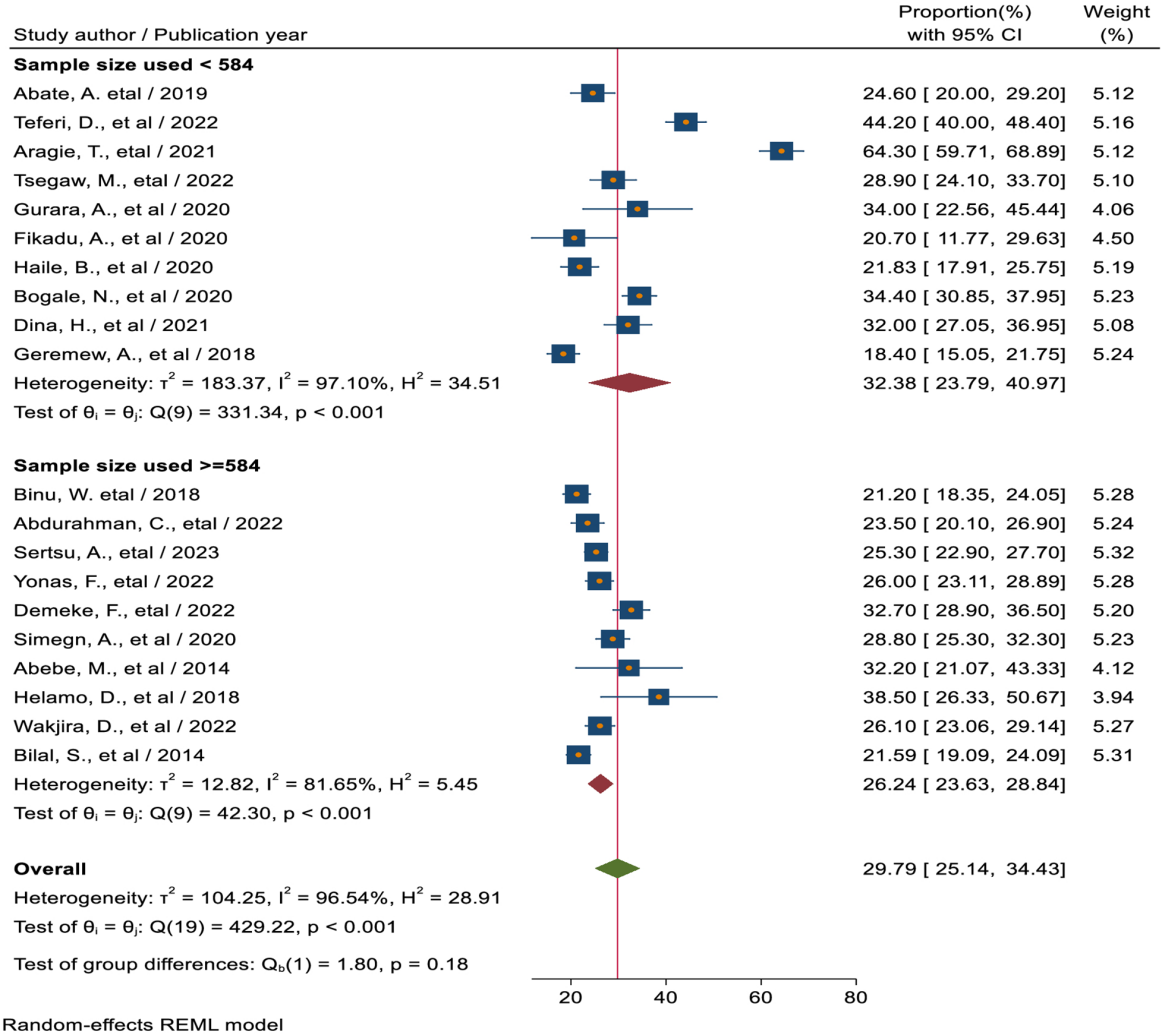
Sub group analysis based on sample size of sexual and reproductive health service utilization among high school students in Ethiopia.

In terms of sample size, the included studies were divided into two groups based on their sample size, with the cutoff point set at the median sample size of 584. Accordingly, in studies with a sample size <584, the pooled magnitude of sexual and reproductive health service utilization was 32.38% (95% CI: 23.79, 40.97). In contrast, it was 26.24% (95% CI: 23.63, 28.84) for studies with a sample size ≥584 (Figure 4).

## Sensitivity analysis

**Figure 5:**
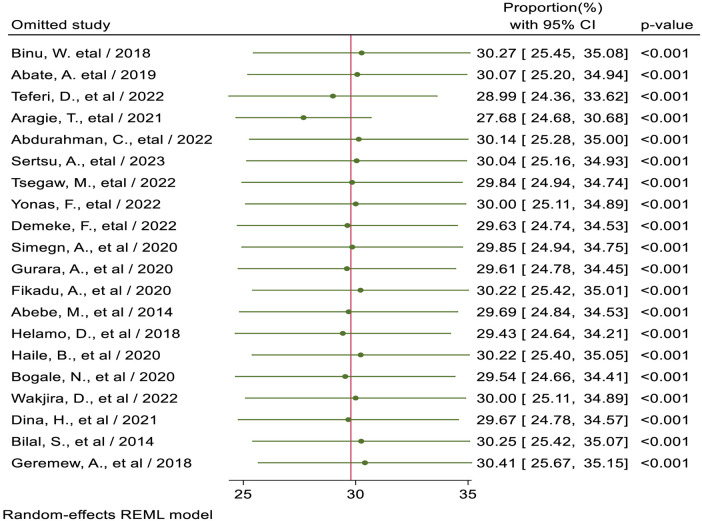
Sensitivity analysis for pooled proportion of sexual and reproductive health service utilization among high school students in Ethiopia.

Sensitivity analysis was conducted to detect each study's effect on the overall prevalence of sexual and reproductive health service utilization among high school students by excluding one study at a time. Based on the findings from sensitivity analysis, no studies in the review impacted the pooled level of sexual and reproductive health service utilization among high school students (Figure 5).

### Publication bias assessment

**Figure 6:**
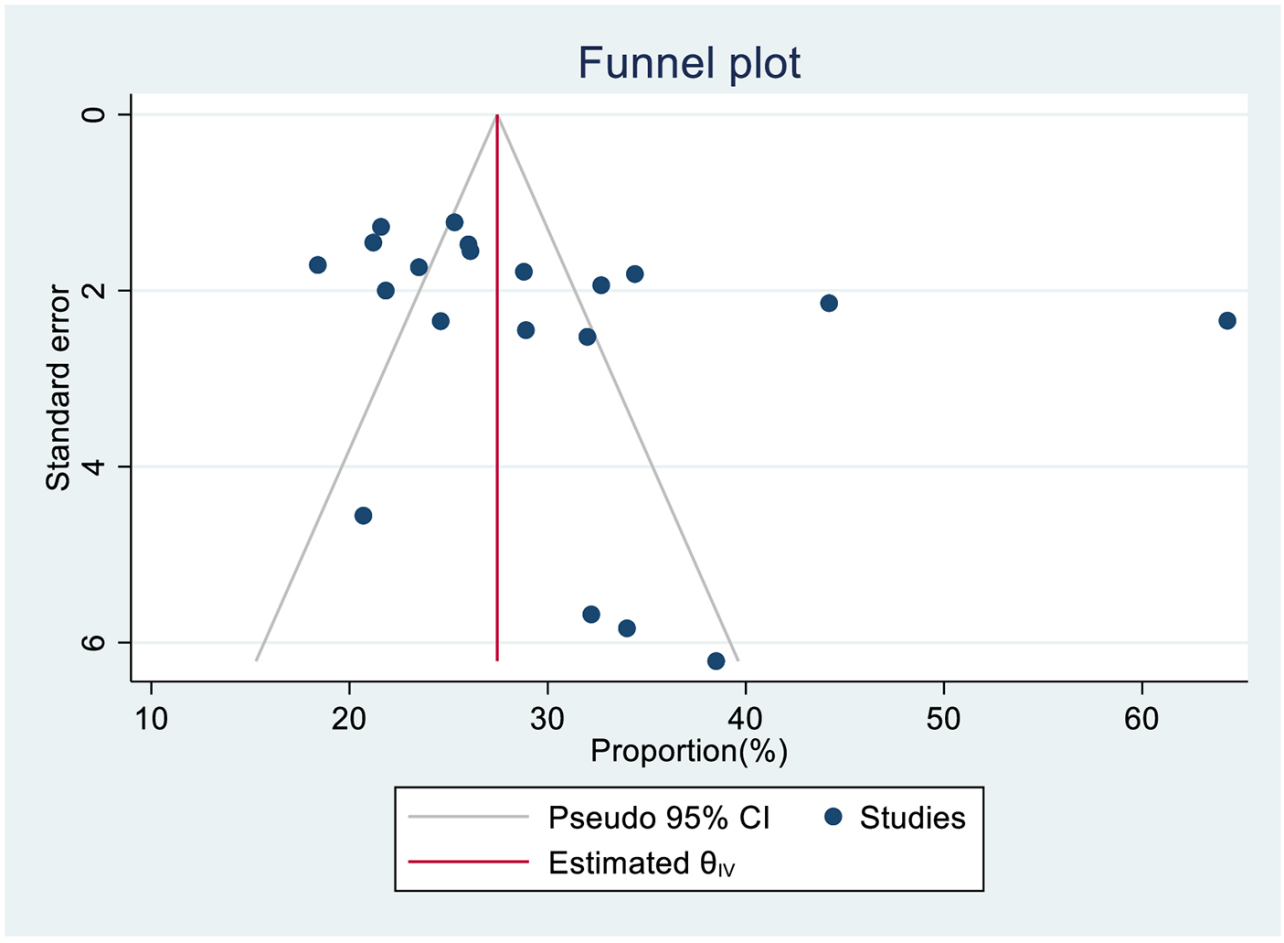
Funnel plot of publication bias assessment for the pooled magnitude of sexual and reproductive health service utilization among high school students in Ethiopia.

A funnel plot (subjectively) and Egger's regression and Begg's test (objectively) assessed publication bias. Based on the funnel plot, the observed proportion of SRHS across 20 studies was nearly symmetrically distributed around the pooled proportion (Figure 6). The *p*-value for regression-based Egger's test was 0.345, indicating the absence of publication bias and the *p*-value for Begg's test was 0.02, indicating the presence of publication bias. We used a trim-and-fill study in the random effects model to reduce the impact of publication bias. The prevalence estimates did not change substantially between the original model and the trim and fill model. Thus, Duval and Tweedie's nonparametric trim and fill analysis did not account for additional studies (Table 3).

**Table 3:** Non-parametric trim and fill analysis of publication bias assessment pooled proportion of sexual and reproductive health service utilization among high school students in Ethiopia.

Studies	Proportion (%)	95% conf. interval	
		Lower confidence interval	Upper confidence interval
Observed	29.785	25.138	34.432
Observed + Imputed	29.785	25.138	34.432

### Factors associated with reproductive health utilization among secondary school students

Students' grade level, age category, knowledge of SRH issues, previous history of sexual intercourse, history of STI, discussion about sexual and reproductive health, and presence of SRH service facility in the school were statistically significant associations with SRH service utilization of high school students. On the other hand, sex, residence, the presence of reproductive health problems, and the presence of nearby health facilities around their living area had no statistically significant association with SRH service utilization of high school students (Table 4).

**Table 4:** Factors associated with SRHSU of high school students in Ethiopia.

Factors	Number of studies included	Pooled odds ratio	Lower 95% confidence interval	Upper 95% confidence interval
Age 20–24 years	3	2.61	1.79	4.14
Grades 11 & 12	3	2.33	1.39	3.81
Higher level of knowledge towards SRHS	7	3.10	1.67	5.77
Previous history sexual intercourse	8	4.18	2.59	6.75
History of STI	3	3.74	2.22	6.31
Ever discussion about SRH issues	2	4.04	1.62	10.03
The presence of an RHS facility in the school	2	2.55	1.72	3.77
Sex (male)	5	1.56	0.88	2.77
Residence (urban)	2	1.46	0.18	11.61
Presence of RH problem	2	2.99	0.76	11.77
The presence of a nearby health facility	2	3.16	0.64	15.73

#### Grade level

**Figure 7:**
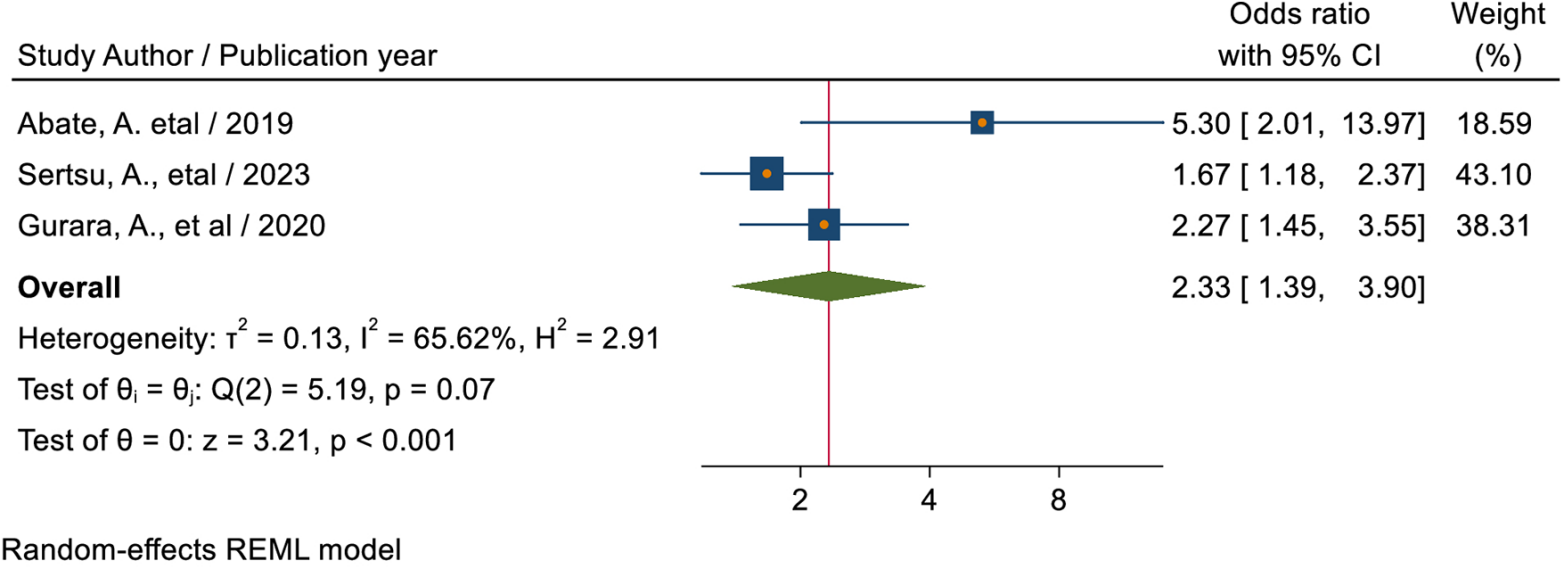
Forest plot for the association between grade level of students and sexual and -reproductive service utilization among high school students in Ethiopia.

The pooled estimate of three studies (40, 42, 48) showed that students who were in grades 11 and 12 had a 2.33-times (AOR = 2.33, 95% CI: 1.39, 3.90) more likelihood of using sexual and reproductive health services than students in grades 9 through 10 (Figure 7).

#### Age category of respondents

**Figure 8:**
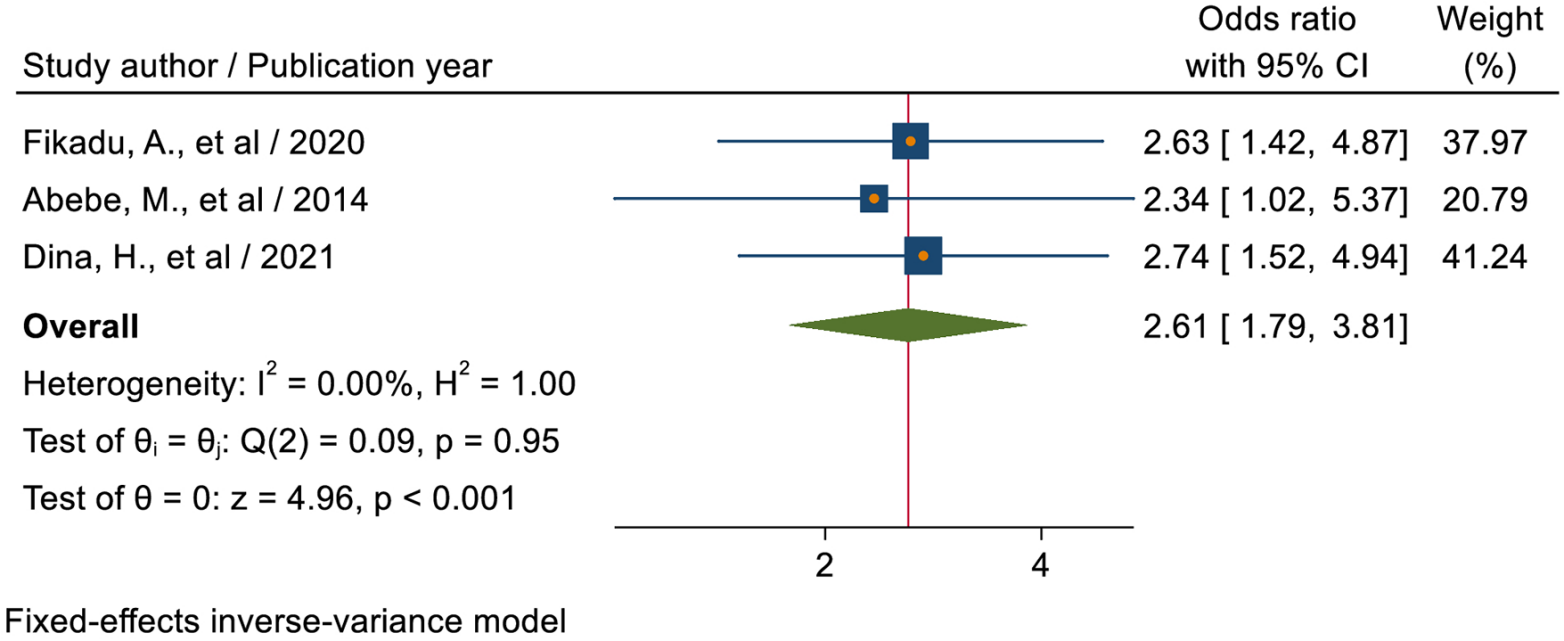
Forest plot for the association between the age category of students and sexual and reproductive health service utilization among high school students in Ethiopia.

The pooled estimate of three studies (21, 43, 50) revealed that students with an age range of 20–24 years were 2.61 times (AOR = 2.61; 95% CI: 1.79–3.81) more likely to utilize sexual and reproductive health services as compared to students with an age range of 15–19 years (Figure 8).

#### Knowledge level of students towards sexual and reproductive health

**Figure 9:**
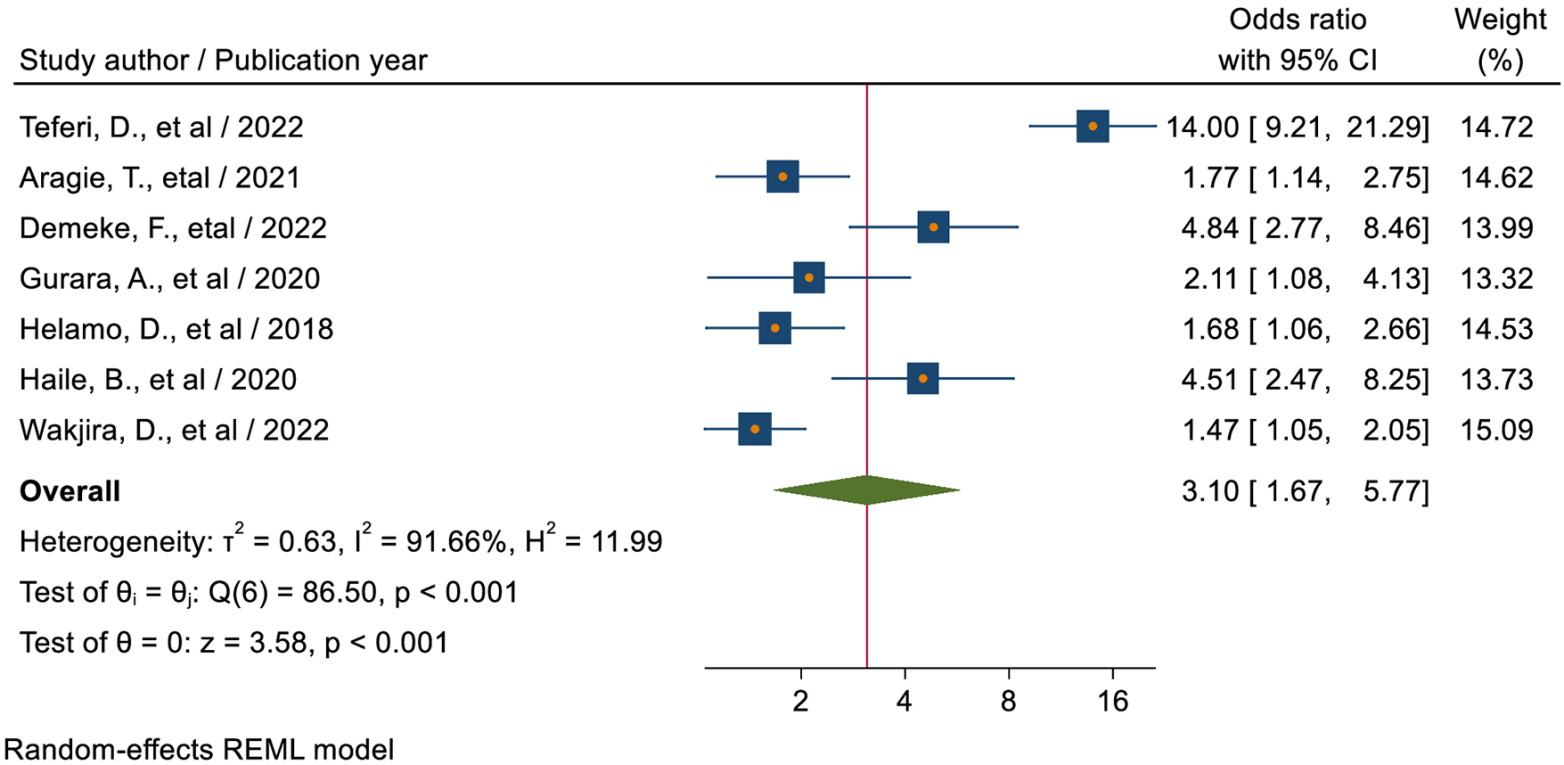
Forest plot for the association between knowledge status towards sexual and reproductive health, and sexual and reproductive service utilization among high school students in Ethiopia.

The pooled estimate of six studies (38, 41, 46, 49, 52, 53) showed that students who had higher level of knowledge regarding SRH service were 3.54 times (AOR = 3.54; 95% CI: 1.81–6.94) more likely to utilize sexual and reproductive health services as compared to students who had low level of knowledge (Figure 9).

#### Previous history of sexual intercourse

**Figure 10:**
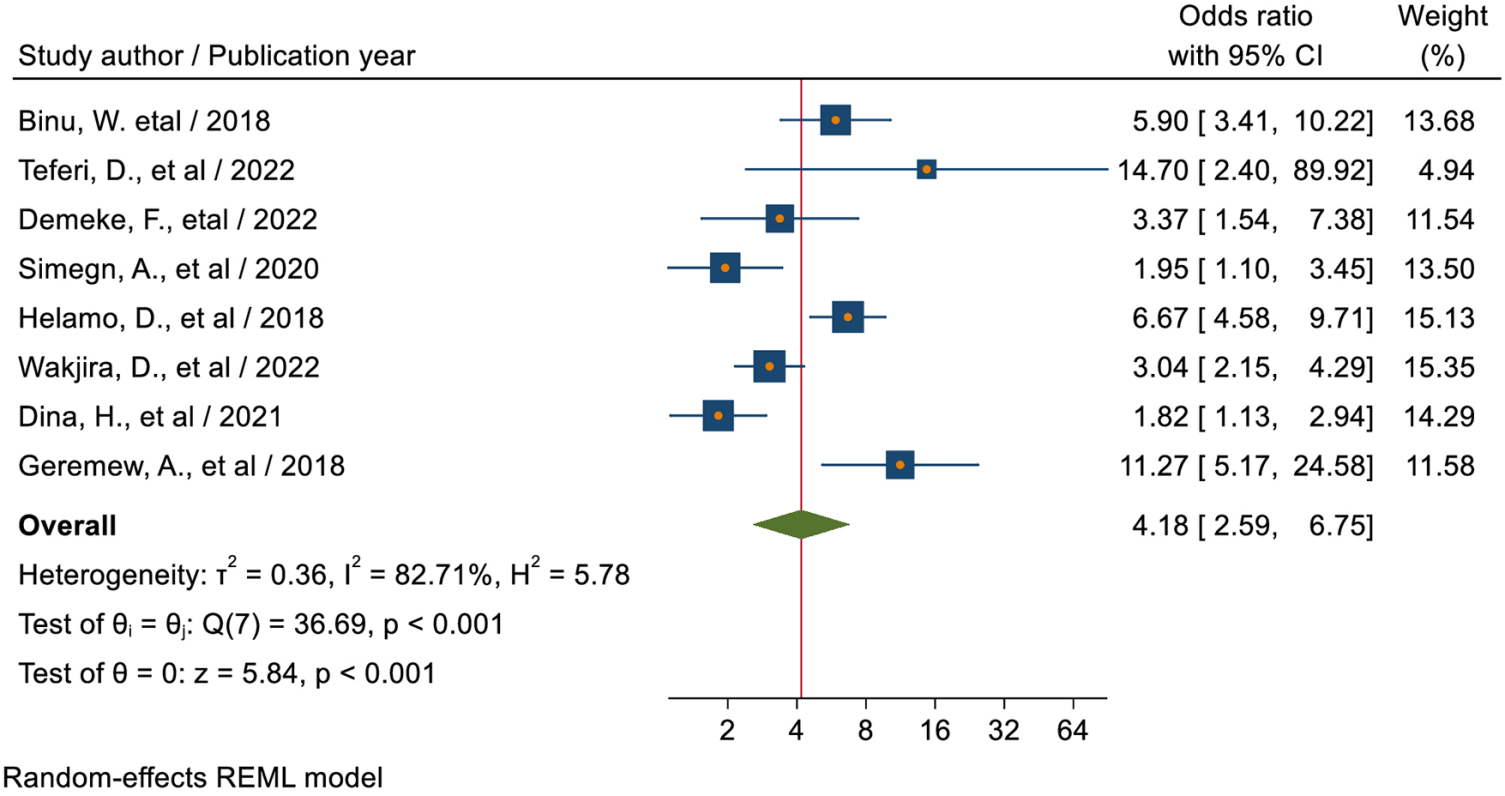
Forest plot for the association between the sexual experience of students and sexual reproductive service utilization among high school students in Ethiopia.

The pooled estimate of seven studies (18, 38, 39, 41, 45, 49, 50, 52) revealed that students who had a previous history of sexual intercourse were 4.53 times (AOR = 4.53; 95% CI: 2.59–7.93) more likely to utilize sexual and reproductive health services as compared to students who had no a previous history of sexual intercourse (Figure 10).

#### Previous history of sexually transmitted infection

**Figure 11:**
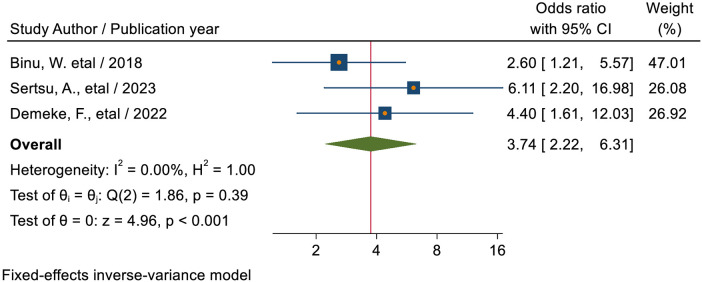
Forest plot for the association between the previous history of sexually transmitted infection of students and sexual and reproductive service utilization among high school students in Ethiopia.

The pooled estimate of three studies (40, 45, 52) showed that students who had a previous history of sexually transmitted infection were 3.74 times (AOR = 3.74; 95% CI: 2.22–6.31) more likely to utilize sexual and reproductive health services as compared to students who had no previous history of sexually transmitted infection (Figure 11).

#### Presence of SRH service facility in school

**Figure 12:**
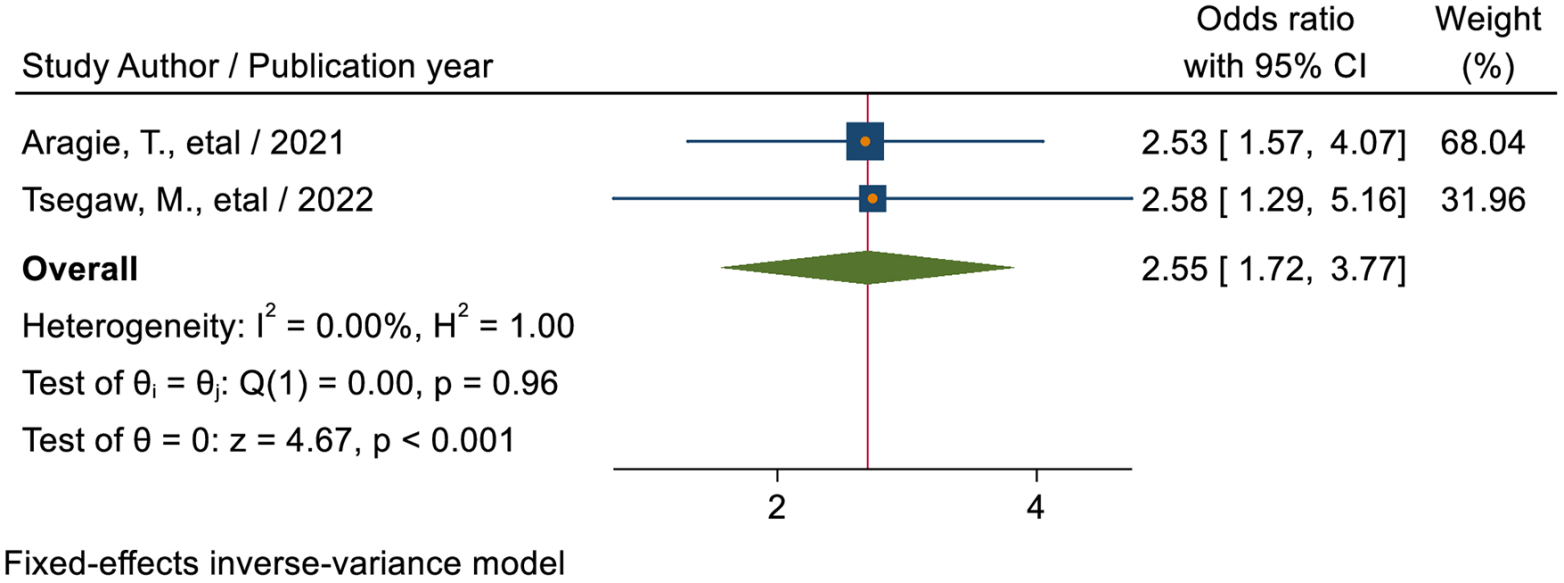
Forest plot for the association between the RHS facility in school and sexual and reproductive service utilization among high school students in Ethiopia.

The pooled estimate of two studies (37, 46) showed that the odds of utilizing SRHS were 2.55 times (AOR = 2.55; 95% CI: 1.72–3.77) higher among students who had RHS facility in their school as compared to students who had no SRH service facility in their school (Figure 12).

#### Discussion on SRH issues sexual and reproductive health service

**Figure 13:**
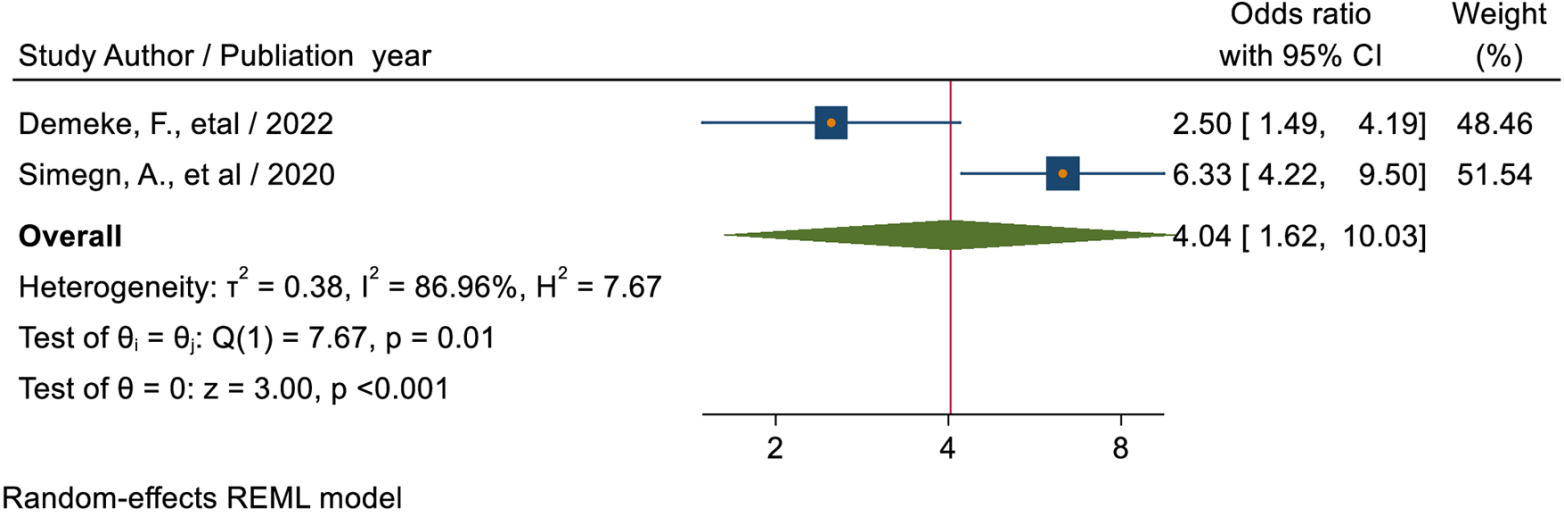
Forest plot for the association between students who had ever discussion on sexual and reproductive health issues, and sexual and reproductive service utilization among high school students in Ethiopia.

The pooled estimate of two studies (39, 52) showed that students who ever discussed reproductive health issues with either healthcare workers families or teachers or peers, or sexual partners were about 4.04 times (AOR = 4.04; 95% CI: 1.62–10.03) more likely to use reproductive health services than those who had not discussed (Figure 13).

## Discussion

The current meta-analysis gives overall evidence on the magnitude of SRHS utilization among high school students in Ethiopia and its associated factors. Accordingly, the pooled magnitude of SRHS utilization among high school students in Ethiopia was found to be 29.79% (95% CI: 25.14, 34.43). This finding was consistent with 30% in Kenya (54). However, it is higher than 6.9% in Malaysia (55), 9.2% in Western Nepal (56), 24.3% in Indonesia (57), and 13.8% in the Philippines (58). However, this finding is lower than previously round as 42.7% in Ethiopia (59), 55.8% in Ghana (60), 38.5% in Kenya (61), and 50.1% in Nigeria (62). This discrepancy may result from variations in study participants' sociodemographic characteristics, cultural influences, availability and accessibility of youth-friendly health facilities, policies and strategies of nations, quality of reproductive health services, and sampling techniques as well as the difference in technological advancement of nations in health information dissemination.

Based on subgroup analysis; the Amhara region had about 32.82% and the Southern Nations, Nationalities, and Peoples' region had 32.22% of high school students utilized SRHS. Whereas about 26.30% of high students in the Oromia region had a relatively lower magnitude of SRH service utilization. This could be due to the variations in the care standards, in the professional and caring ways that sexual and reproductive health care services are provided, and as well as the difference in experiences of the providers.

According to the present meta-analysis, students' grade level, age category, knowledge of SRH issues, sexual experience, history of STI, discussion about sexual and reproductive health issues with others, and presence of SRH service facility in the school were statistically significant association with SRHS utilization of secondary school students.

This study revealed that the pooled estimate of students in grades 11 and 12 had a 2.33-times (AOR = 2.33, 95% CI: 1.39, 3.90) more likely to use sexual and reproductive health services than students in grades 9 through 10. This finding is supported by studies conducted in Kenya (54) where educational level differences are significantly associated with SRH Service utilization. This finding is also supported by another systematic review meta-analysis conducted in Ethiopia (59). Possible causes for this include secondary behavioral changes as students move up the grade levels and increased sharing of SRH services information. This could mean that in comparison to students in grades 9 and 10, grade level 11–12 students had greater access to information. This could also be due to students in grades 11 and 12, who are probably more mature, knowledgeable, and inclined to use the services than students in lower grades. One explanation for this could be that individuals with better educational status were more likely to freely share SRH knowledge with friends, family, and groups, and as they grew older, so did the need for SRH services (63).

In this study, students with an age range of 20–24 years were 2.61 times (AOR = 2.61; 95% CI: 1.79–3.81) more likely to utilize sexual and reproductive health services as compared to students with an age range of 15–19 years. This finding is supported by studies conducted in Kenya (54). This is presumably because, in comparison to youths between the ages of 15 and 19, older youths implement what they have learned about youth reproductive health services and are more inclined to use the SRH programs. They participate in sex as their age increases and exhibit more sexually mature behaviors. To prevent HIV infection and unintended pregnancy, the likelihood of utilizing SRH services increased (20).

Students who had higher level of knowledge regarding SRHS were 3.10 times (AOR = 3.10; 95% CI: 1.67–5.77) more likely to utilize sexual and reproductive health services as compared to students who had lower level of knowledge. It was higher than a study conducted among senior high school students in Indonesia, which found that students who had higher level of SRHS knowledge were 1.74 times more likely to utilize SRHS as compared to students who had lower level of SRHS knowledge (57). It was also supported by a study conducted in Nigeria (64), which states that the majority of youths who used the SRHS services were knowledgeable about SRHS. This is because people who are more knowledgeable about issues related to sexual and reproductive health will grow to be skilled health-seekers. Before being able to quickly and efficiently utilize appropriate treatments, students must have a solid understanding of sexual and reproductive health services. It might also be because responders with a high degree of SRH service knowledge will be aware of the advantages of utilizing SRH services as well as the drawbacks of not using them.

Students who had a previous history of sexual intercourse were 4.18 times (AOR = 4.18; 95% CI: 2.59–6.75) more likely to utilize sexual and reproductive health services as compared to students who had no previous history of sexual intercourse. This finding is lower as compared to the study conducted in Nepal (56). This could be because many sexual and reproductive health services are accessed when young people feel that there are hazards to their reproductive health associated with having sex. This could be because people who have had sex are more likely to seek medical attention and fear negative health consequences like acquiring STIs, getting pregnant unintentionally, and having an abortion. This could also be explained by the fact that young people who had sex were more likely to experience issues related to their reproductive health, which could lead to a greater demand for RH services. This suggests that for young people to engage in safe and healthy behavior, they must have access to a variety of health-related resources, including information and services. Ever engaging in sexual activity was highly correlated with using youth-friendly SRH services, particularly when it came to difficulties from pregnancy. Because women who engage in sexual activity are more likely to become pregnant unintentionally, have STIs, get HIV, and miss school as a result of pregnancy-related issues, they frequently use health facilities for voluntary HIV testing, family planning, and counseling.

Students who had a previous history of sexually transmitted infection were 3.74 times (AOR = 3.74; 95% CI: 2.22–6.31) more likely to utilize sexual and reproductive health services as compared to students who had no previous history of sexually transmitted infection. This could be explained by the possibility that patients with a history of STIs could visit a healthcare facility where they would receive RHS components in addition to STI therapy. This could also be explained by the fact that young people who encountered SRH problems were more concerned about their health when those problems arose. This suggests that for young people to engage in safe and healthy behavior, they must have access to a variety of health-related resources, including information and services.

Students who ever discussed reproductive health issues with either health care workers, families, teachers, peers, or sexual partners were about 4.04 times (AOR = 4.04; 95% CI: 1.62–10.03), compared to those who had not discussed, more likely to use reproductive health care. This finding is supported by a study conducted in Ethiopia (59) and the United States of America (65). This could also be a result of discussions helping young people learn pertinent information about various sexual and reproductive health concerns and services that are accessible. Because of this, knowledgeable young people might be more likely to utilize these services. This could also be because dialogue enables young people to learn pertinent information about various health concerns and resources that are offered. Youth who are well-informed may therefore be more likely to make the wise choice to use the programs (66). This might be explained by the confidence that was built up through a conversation about SRH difficulties with family and friends and the notable differences that resulted from that conversation. Moreover, this may be because candid conversations about SRH issues between families and children raise awareness and help people feel less self-conscious and afraid of being noticed when receiving SRH services. Also, the conversation opens up more opportunities for youth to exchange SRH knowledge and firsthand experiences with health-related issues. This could lead to improved youth awareness of SRH services and the development of favorable attitudes towards youth friendly reproductive health services, which could encourage young to utilize such services.

The odds of utilizing SRHS were 2.55 times (AOR = 2.55; 95% CI: 1.72–3.77) higher among students who had an SRH service facility in their school as compared to students who had no SRH service facility in their school. This is lower than a study conducted in Nepal, 14.85 (56) which stated that students attending secondary schools that included SRH in the school's health services were 15 times more likely to utilize SRH services. This is most likely because the respondents who have access to health facilities in their school are likely to be exposed to health education regarding sexual and reproductive health services during their visits, allowing them to learn about the different types, advantages, and availability of sexual and reproductive health services.

## Limitation of study

Despite adhering to PRISMA guidelines throughout the review process, this review had the following limitations. We only include an observational study design for our review. We may miss qualitative and experimental studies. The other possible drawback of the current systematic review could be that it only included full-text publications written in English, which means important works that may have been written in other languages on the subject were missed.

## Conclusion

The overall magnitude of sexual and reproductive health service utilization among high school students was low in Ethiopia. Students with age range between 20 and 24 years, grades 11–12, higher level of knowledge regarding sexual and reproductive health services, history of sexual experience, history of sexually transmitted infection, presence of reproductive health service facility in the school and ever discussed about sexual and reproductive health issues had positive a statistically significant association with sexual and reproductive health service utilization as compared to their counterparts. Thus, policymakers and program implementers had better to enhance sexual and reproductive health service utilization among high school students, attention should be given to the identified determinants. It is also important for schools to provide easily accessible sexual and reproductive health services that are affordable, confidential, and convenient for high school students, including follow-up care options.
